# Towards Real-Time Prediction of Freezing of Gait in Patients With Parkinson’s Disease: Addressing the Class Imbalance Problem

**DOI:** 10.3390/s19183898

**Published:** 2019-09-10

**Authors:** Nader Naghavi, Aaron Miller, Eric Wade

**Affiliations:** Department of Mechanical, Aerospace and Biomedical Engineering, University of Tennessee, Knoxville, TN 37996, USA; amill106@utk.edu (A.M.); erwade@utk.edu (E.W.)

**Keywords:** ensemble classifier, data synthesis, ADASYN, cost of classification, freezing of gait, Parkinson’s disease, wearable sensors

## Abstract

Freezing of gait (FoG) is a common motor symptom in patients with Parkinson’s disease (PD). FoG impairs gait initiation and walking and increases fall risk. Intelligent external cueing systems implementing FoG detection algorithms have been developed to help patients recover gait after freezing. However, predicting FoG before its occurrence enables preemptive cueing and may prevent FoG. Such prediction remains challenging given the relative infrequency of freezing compared to non-freezing events. In this study, we investigated the ability of individual and ensemble classifiers to predict FoG. We also studied the effect of the ADAptive SYNthetic (ADASYN) sampling algorithm and classification cost on classifier performance. Eighteen PD patients performed a series of daily walking tasks wearing accelerometers on their ankles, with nine experiencing FoG. The ensemble classifier formed by Support Vector Machines, K-Nearest Neighbors, and Multi-Layer Perceptron using bagging techniques demonstrated highest performance (F1 = 90.7) when synthetic FoG samples were added to the training set and class cost was set as twice that of normal gait. The model identified 97.4% of the events, with 66.7% being predicted. This study demonstrates our algorithm’s potential for accurate prediction of gait events and the provision of preventive cueing in spite of limited event frequency.

## 1. Introduction

Parkinson’s disease (PD) is clinically characterized by both motor and non–motor symptoms. The most common motor symptoms are slowness of movement (bradykinesia), hastening of the gait (festination), paucity of spontaneous movements (akinesia), and poor postural stability. Gait impairment is the most incapacitating symptom among patients with PD [1], as it negatively affects mobility and independence and results in fall-related injuries, emotional stresses, and deterioration of patients’ quality of life [2,3,4,5].

Freezing of gait (FoG) is commonly regarded as a feature of akinesia, an extreme form of bradykinesia [6]. FoG is described as brief episodes of inability to step forward or as taking extremely short steps when initiating gait or turning [7]. FoG is highly affected by environmental stimuli, cognitive input, medication, and anxiety [8,9]. It occurs more frequently at home than in the clinic, in complete darkness, and in other settings that require greater cognitive load like dual-tasking situations [10,11,12,13].

### 1.1. FoG Treatment

PD treatments have been under investigation for some time, with levodopa (LD) and dopamine agonist (DA) as the most common pharmacological treatments for patients suffering from impaired activities of daily living. Although LD decreases duration of FoG episodes and their frequency during on-medication periods, FoG incidents are still difficult to treat during off state and in advanced stages of the disease. On the other hand, drugs for non-motor symptoms can interfere with the effectiveness of LD and aggravate motor symptoms [14]. DA, in contrast with LD, may provoke more FoG episodes in early stages of disease [15]. For many patients with concurrent FoG symptoms and cognitive disorder, the efficacy of medication therapy is poor and deep brain stimulation (DBS) is often prescribed [16]. Non–randomized studies with low sample sizes have shown that DBS can improve FoG and the effect can last for at least 1 year, however, the risk of aggravating other symptoms still exists [16,17]. Therefore, new effective non-pharmacologic treatments are still needed to relieve FoG symptoms.

### 1.2. External Cueing

It is thought that motor dysfunctions in PD result from limited resources and less automaticity of motor plans due to the damage to the basal ganglia [18]. To tackle this, non-invasive, non-pharmacological interventions in the form of external stimuli have recently gained attention. Patients are instructed how to shift their attention toward gait using external cues as discrete targets [19,20]. Spatial cues (e.g., strips placed or laser beams projected on the floor) can be customized for each patient based on their stride length to show patients *where* to put their next step. On the other hand, temporal cues (e.g., auditory metronome or vibrotactile feedback) are customized based on cadence and inform users *when* a step should be taken. Studies suggest that externally cued training can reduce FoG severity and improve gait velocity, stride length and upper-limb movements immediately after training [19,21,22].

Frazzitta et al. investigated the effects of visual and auditory external cueing on PD patients with FoG symptoms. Patients received cueing therapy daily for 20 min and demonstrated statistically significant improvements in Freezing of Gait Questionnaire (FOGQ) score after four weeks [23]. Nieuwboer et al. delivered cueing training in the home of 153 individuals with PD. Cueing devices provided three cueing modalities: (1) auditory (a beep triggered through an earpiece); (2) visual (light flashes triggered through a light-emitting diode attached to a pair of glasses); and (3) somatosensory (pulsed vibrations triggered by a miniature cylinder worn under a wristband). The results showed that severity of freezing was reduced by 5.5% in patients with FoG symptoms [24]. Kadivar et al. compared a battery of clinical assessments after a 6-week training session and 4 weeks follow-up in two groups of eight patients practicing with rhythmic auditory stimulation stepping (RAS group) and no-cue stepping (no RAS group). Results suggested that the RAS group significantly improved FoG symptoms (as measured by FOGQ) and maintained improvements above baseline values for at least 4 weeks after practice termination [25].

### 1.3. FoG Detection

“Always-on” cueing is defined as a paradigm in which stimulus is delivered repeatedly to the user regardless of any prior or imminent FoG episodes. However, individuals with PD are known to adapt to interventions provided continuously, thus reducing the effect of cueing [19,26]. Therefore, it is ideal to deliver an external stimuli only when it is contingent on symptom onset. This requires the development of an integrative system capable of automatically detecting FoG episodes. A variety of methods for such an approach include using data captured from electrocardiography (ECG) systems [27], electromyography (EMG) systems [28,29], 3D motion systems [30,31], foot pressure sensors [32,33], and Inertial Measurement Units (IMUs) [34,35].

To date, a variety of approaches have been employed resulting in varied classification accuracy. Example applications include that of Tahafchi et al., who used temporal, spatial, and physiological features to train a Support Vector Machine (SVM) classifier to identify freezing episodes. Data were collected using inertial sensors attached to the thigh, shank, and foot, and non-invasive surface EMG sensors attached to quadriceps/tibialis muscles of PD patients. They detected 90% of the FoG events correctly, while identifying 8% of the non-FoG data incorrectly as FoG [36]. Another group, Mazilu et al., tested different supervised machine learning algorithms on detecting FoG events using 3-D acceleration signals collected from the ankle, knee, and hip of ten PD patients. A correlation-based feature subset selection was used to choose only the most discriminative features. They compared results from two different approaches: “patient-dependent”, in which both training and testing data were from the same participant, and “patient-independent” utilizing leave-one-out cross validation. Their results for patient-dependent models showed average sensitivity, specificity, and F1 (see Equation (4)) of 99.54%, 99.96% and 99.75%, respectively, using Random Forest classifiers. However, the average performance for patient-independent models resulted in much lower sensitivity and specificity (66.25% and 95.38%, respectively) [37].

Using more recent techniques, Camps et al. applied a deep learning (DL) method to detect FoG episodes in home environments. Their algorithm employed an eight-layered one-dimensional convolutional neural network and spectral window stacking as data representation to combine information from both the prior and current signal windows. They used data from a single IMU placed on the waist of thirteen patients to train the DL model and tested the model on data from four other patients (not included in the training set). The DL model detected FoG episodes with sensitivity and specificity of 91.9% and of 89.5%, respectively [38]. Finally, Xia et al. implemented a deep convolutional neural network to detect FoG events. The system segmented 1-dimensional acceleration signals into windows of 4 s and realized automatic feature learning in order to discriminate FoG from normal gait, thus, removing the need for extracting hand-crafted features and time-consuming feature selection. They reached average sensitivity and specificity of 99.64% and 99.99%, respectively, using patient-dependent, and 74.43% and 90.60% using patient-independent models [39].

The described studies achieved high classification accuracy for FoG detection, especially with patient-dependent models which reduces the effect of heterogeneity in data from different participants. However, these studies seldom reported the detection latency (i.e., time associated with classification after FoG onset) and prediction capability (i.e., time associated with classification prior to FoG onset). This study aimed to evaluate the classification performance of individual and ensemble classifiers for FoG, while also addressing the class imbalance problem inherent to FoG (i.e., the relative infrequency of FoG occurrence when compared to normal gait behaviors). With the encouraging results obtained in the experiments, we hope this study can provide an effective intervention to accurately predict FoG events using wearable inertial sensor data, and ultimately help patients prevent FoG through external cueing.

### 1.4. FoG Prediction

Providing cues during an actual FoG episode may result in cognitive overload by superimposing an external stepping rhythm, which may aggravate the FoG. Ginis et al. suggested that an optimal timing for delivering intelligent cues is before the actual onset of a potential FoG episode [19]. Such predictions would also enable preventive cueing and potentially reduce the likelihood of this disabling symptom [24]. Palmerini et al. trained a linear discriminant analysis classifier to discriminate pre-FoG episodes from normal gait in eleven PD patients using a wearable multi-sensor setup. After removing data corresponding to FoG and with no sufficient motion, data for each patient was divided into 2 s windows of pre-FoG and normal gait. The classifier identified 83% of the pre-FoG episodes on average in patient-dependent model [40].

Torvi et al. developed a deep learning algorithm to predict FoG events before their occurrence. They also studied the performance of transfer learning algorithms to address the domain disparity between data from different subjects, in order to develop a better prediction model for a particular subject. The model predicted 88% of the events within 1 s before FoG occurrence in patient dependent mode, with 50% of the data used for training. The prediction accuracy improved to 93% with the addition of transfer learning techniques to develop a prediction model for a particular subject by leveraging data from different subjects [41].

Existing literature reported wide ranges of FoG detection and prediction accuracy for participants using primarily patient-dependent approaches. This is partly due to the fact that participants reacted differentially to the FoG stimuli included in the experiments, which caused wide ranges of FoG to non-FoG data ratios. Oftentimes, high levels of class imbalance in data set aggravated model performance. Therefore, new techniques are needed to address this issue, particularly when patient-dependent models are to be developed and implemented in cueing intervention devices. In this study we investigated the effect of data imbalance on the performance of classifiers in predicting and detecting FoG. We applied three common approaches: using ensemble classifiers [42], adding new synthetic FoG samples to the training dataset to improve balance [43], and increasing misclassification cost for the minority class, i.e., FoG [44]. The selected classifier must have high performance in discriminating FoG from normal gait within an appropriate time period before or after FoG occurrence.

## 2. Materials and Methods

### 2.1. Experiment Protocol

**Figure 1:**
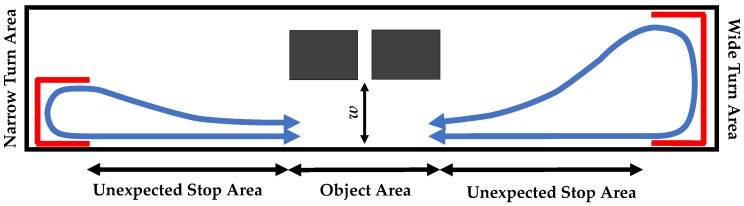
Experiment layout. Number of obstacles in the object area varied between 0, 1 and 2. The width of walking path in the object area (*w*) varied between 150% and 100% of shoulder width of participants.

Schaafsma et al. described five subtypes of freezing triggerred by gait initiation, turning, walking through tight hallways, terminating gait, and open space hesitation [45]. In order to trigger FoG symptoms, our experiment protocol included turns within wide and narrow turning areas at either end of a hallway, varied path widths (using shelves in the middle of the hallway), expected stops before turns and unexpected stops instructed vocally when walking straight (Figure 1). Acceleration data in two axes of horizontal forward (perpendicular to the frontal plane) and vertical (perpendicular to the transverse plane) were captured using two accelerometers (APDM Inc. Opal Sensors, Portland, OR, USA, www.apdm.com) placed on left and right ankles superior to the tibia/talus joint. Data were sampled at 128 Hz and stored on the sensors’ internal memory. As the algorithm will be implemented in an online cueing device, we tested the feature extraction methods on an Android phone using a researcher developed application. The results suggested that, due to the limited computation power of cellphones compared with PCs, the data should be down-sampled to reduce the computation time for feature extraction. Thus, the sampling rate of the collected data was then reduced to 64 Hz using linear resampling.

### 2.2. Dataset

Eighteen participants with PD (12 M/6 F, 70.0 ± 8.7 years, Hoehn and Yahr score between 2 and 4) were recruited and screened according to the standards of the University of Tennessee Institutional Review Board (IRB). Participants walked in a narrow hallway (12 m × 1.5 m) for 29.1 ± 8.2 min (524 min in total). 156 FoG episodes were captured over 18.4 min (2.0 ± 2.2) from nine participants.

An experienced clinician determined onset and offset of FoG episodes and labeled acceleration signals using captured videos. The onset of a FoG episode was detected when the normal gait pattern (i.e., alternating left–right stepping) was arrested, and the end of the episode was marked as the time when the pattern was resumed. The remaining data were labeled as normal gait, stops, and no-activity periods. Stop labeled data were periods during which the participants were asked to stop as part of the experiment, while no-activity periods included data not specific to the experiment, e.g., when the sensors were installed on the user’s body or the user is performing activities unrelated to the experimental protocol such as resting. If the no-activity and stop periods took longer than 2 s and 1 s, respectively, the corresponding windows were excluded to create a binary class dataset of normal gait and FoG. Data from 1 second pre-FoG periods (not labeled as “no-activity” or “stop”) were also labeled as FoG in order to enable the system to predict FoG before its actual onset.

### 2.3. Feature Extraction

**Figure 2:**
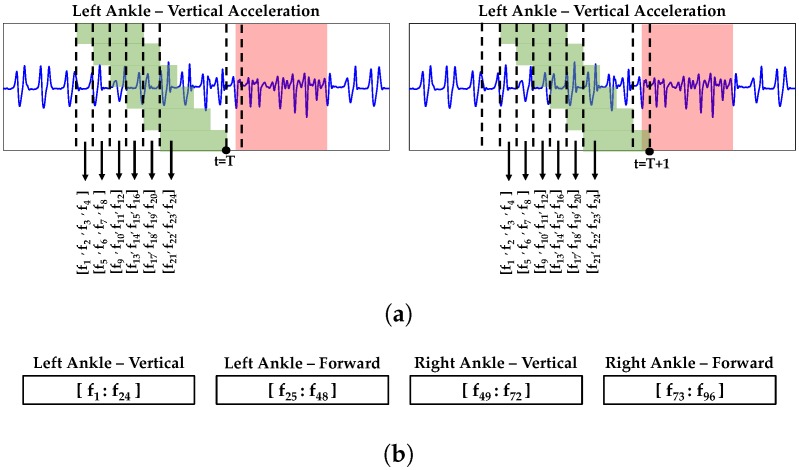
The process of creating samples from acceleration signals. (**a**) Extracting features from six successive windows at time t = *T* (left) and the next time step, t = T+1 (right). Red highlighted area shows FoG labeled period using recorded videos, green boxes show length of windows used to extract features from acceleration signal; (**b**) Combining arrays of features from different combinations of sensor-axis.

In order to extract quantitative feature from continuous sensor data, we used sliding windows of 2 s with 75% overlap to extract four features from acceleration data (Table 1, Figure 2). These four features indicated best performance on classification of gait using the collected data from the participants. We showed prior that FoG is a dynamic process and using features from multiple successive windows can improve FoG detection [46]. In this study, sample $s_{T}$ in the dataset was formed by feature values from the current window (t=T) and five previous windows. Figure 2a demonstrates how an array of 24 elements is formed for vertical acceleration of left ankle (four features extracted from each of six successive windows). For each sensor-axis combination, the array of 24 elements is created and appended to form a sample with 96 elements at time *T* (Figure 2b). For the next time step (t=T+1), the six windows are moved forward one step (0.5 s) and the same process is followed to form sample $s_{T+1}$. The sample is labeled as FoG if at least one FoG labeled timestamp is included in the window nearest to the current time (the right most window). In Figure 2a sample $s_{T}$ is an example of normal gait, while $s_{T+1}$ is an instance of FoG.

### 2.4. Data Synthesis

Synthetic Minority Over-Sampling Technique (SMOTE) is an algorithm to improve class balance and mitigate over-fitting by creating new synthetic samples from the minority class using linear interpolation between existing minority class samples [48]. ADAptive SYNthetic (ADASYN) is an extension that creates samples of the minority class in the vicinity of the boundary between the two classes [43]. ADASYN uses a density distribution function to identify the samples of minority class close to the boundaries and determine the number of synthetic samples to be generated in the neighborhood of each sample [49]. The number of required synthetic samples is specified by:

(1) $$G=(|S_{maj}|-|S_{min}|)\times \beta$$

where $|S_{maj}|$ and $|S_{min}|$ are the number of samples in the majority and minority classes, respectively, and βϵ[0,1] specifies the desired balance level after the synthetic data have been added to the dataset. β=0 means no new data will be created, and β=1 creates an absolute balanced dataset where both classes form 50% of the entire dataset.

The value of β was changed (βϵ[0,0.2,0.5,1]) to investigate the effect of dataset imbalance on classifiers performance. For the original dataset, all classifiers were trained and tested using 60% and 40%, respectively, of all data. For the ADASYN synthesized dataset, the same training data were used to generate new synthetic samples which were then added to the training set. The same testing set was used to evaluate model performance with no synthetic samples included in the testing set. We used MATLAB (Release 2018b, The MathWorks, Inc., Natick, MA, USA) to generate new synthetic samples and train/test the classifiers.

### 2.5. Misclassification Cost

We also investigate the misclassification cost, *C*, which quantifies the importance of incorrectly classifying samples from one class as the other. Cost-sensitive classification incorporates fixed and unequal misclassification costs between classes in decision-making. Each element $\lambda_{ij}$ in the cost matrix represents the cost of classifying a sample from a true class *j* to class *i*. The diagonal elements are usually set to zero, meaning correct classification has no cost. The adjusted prior probability for class *i* in a binary dataset is defined as:

(2) $$\tilde{P_{i}}=\lambda_{ij}\times P_{i}$$

where $\tilde{P_{i}}$ and $P_{i}$ are adjusted prior probability and prior probability, respectively, for class i=1,2. In this study we chose normal gait to be class 1 and FoG to be class 2. Then we set the cost matrix to:

(3) $$C=\left[\begin{array}{cc} 0 & C_{NG}\\ C_{FoG} & 0 \end{array}\right]$$

where $\lambda_{12}=C_{NG}$ is the cost of misclassifying a sample from normal gait as FoG, and vice versa for $\lambda_{21}=C_{FoG}$. As it is of essential importance to detect as many FoG episodes as possible, the misclassification cost of FoG instances was set equal or greater than one ($C_{FoG}\;\epsilon \;\left[1,2,3\right]$), while keeping the cost for normal gait class equal to one in all cases ($C_{NG}=1$). $C_{FoG}>3$ showed poor performance in preliminary results and was excluded from analyses here.

### 2.6. Classifiers

Ensemble analysis is a method which is commonly used in many data mining problems such as classification [50], clustering [51], and anomaly detection [52] in order to reduce the dependence of the model on the specific data set or data locality. This greatly increases the robustness of the data mining process. In this study, we trained k-Nearest Neighbors (kNN), Support Vector Machine (SVM) and Multilayer Perceptron (MLP) classifiers to detect FoG. We also used boosting and bagging methods to train ensemble classifiers formed by these individual classifiers to distinguish between normal gait and freezing episodes (ClsfBoosting and ClsfBagging, respectively). Finally, we trained ensemble classifiers of decision trees trained using specified ensemble techniques (AdaBoost, TreeBaggers, and RandomForest) [50].

### 2.7. Performance Measures

Table 2 shows the confusion matrix for the binary classification of gait (normal gait (NG) and freezing of gait (FoG)). We used sensitivity, specificity, and F1 as performance measures to compare classifiers:

(4a) $$\begin{array}{c} \mathrm{Sensitivity}=TP/(TP+FN) \end{array}$$

(4b) $$\begin{array}{c} \mathrm{Sensitivity}=TN/(TN+FP) \end{array}$$

(4c) $$\begin{array}{c} F1=2TP/(2TP+FP+FN) \end{array}$$

### 2.8. Data Analyses

In the next section, we presented the results for the three performance measures of selected classifiers for patient-dependent and -independent models. The sensitivity and specificity demonstrate the accuracy of the models in classifying FoG and normal gait classes, respectively. F1, on the other hand, shows the overall performance of the models in classifying both classes and is used as the main performance measure to select the model with highest performance. Finally, we investigated FoG detection latency and prediction capability for the classifier with highest F1 in patient-dependent experiments. The results show how many of the events were predicted within 2 s before FoG onset, and how many were detected within 4 s after FoG occurrence.

## 3. Results

### 3.1. Patient-Dependent Models

In patient-dependent models, we used data from seven participants who froze more frequently during the experiments (22±12.6 events). The other two participants who experienced FoG during the experiments, froze only once during the experiments and, as the episodes were relatively short, were excluded from further analysis. Table 3 shows the average performance of patient dependent models using the original imbalanced dataset. Among all the individual and ensemble classifiers, SVM showed highest sensitivity (85.6%), and ClsfBagging demonstrated highest specificity and F1 (95.8% and 87.7%, respectively). The results suggest that using ensemble classifiers led to improved FoG detection, measured by F1 as the overall performance measurement.

**Figure 3:**
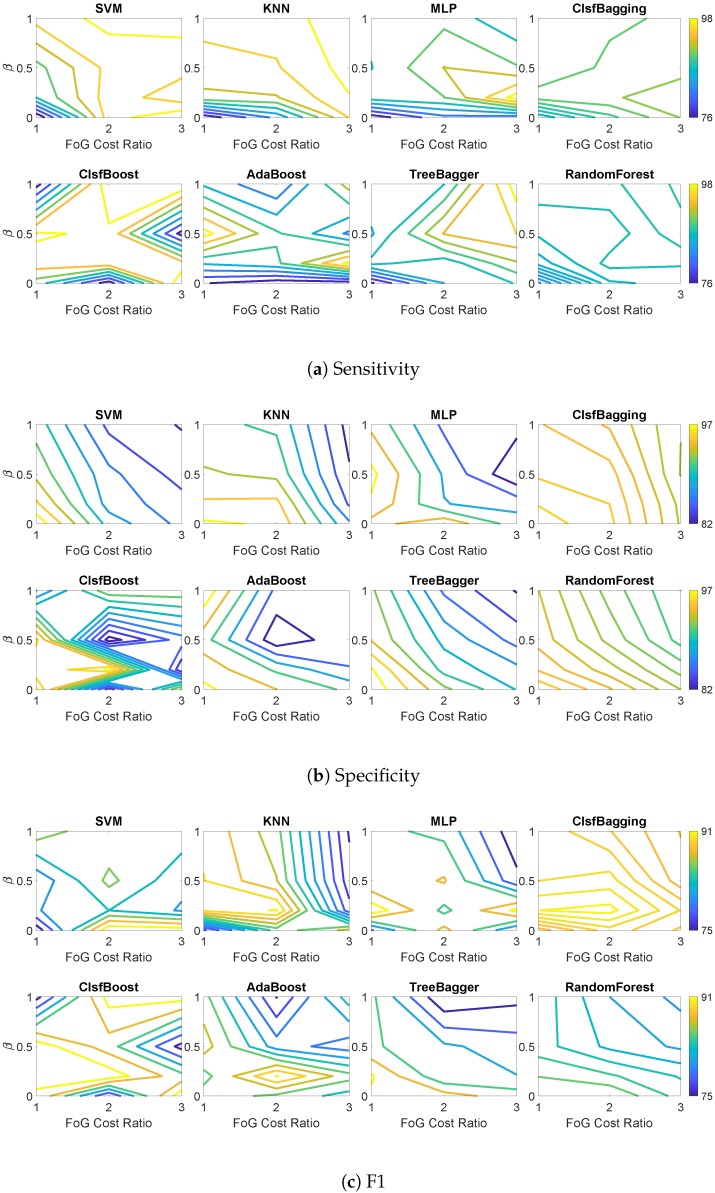
Performance measures of classifiers for patient-dependent models using synthetic data and cost of misclassification.

In order to see the impact of data imbalance on classifier performance, we changed the β value in ADASYN from zero (no synthetic data added to the training set) to one (creating a fully balanced training dataset). We also changed the ratio of cost between two classes (cost of FoG to normal gait) from one to three to investigate the effect of misclassification cost on performance of classifiers. Figure 3 shows how sensitivity, specificity, and F1 of classifiers changed by using different levels of imbalance and cost ratio. Table 4 shows the average performance of classifiers and their best results for the three performance measures in patient dependent models. Among all classifiers and combinations of β and cost ratios, KNN showed highest sensitivity (97.6%) and specificity (96.2%), and ClsfBagging had the highest total performance measured by F1 (90.7%). The results show that using data synthesis and increased cost of misclassification, improved sensitivity of ClsfBagging from 85.2% to 90.8%, while keeping the specificity almost untouched, which resulted in improved F1 from 87.7% to 90.7%.

### 3.2. Patient-Independent Models

**Figure 4:**
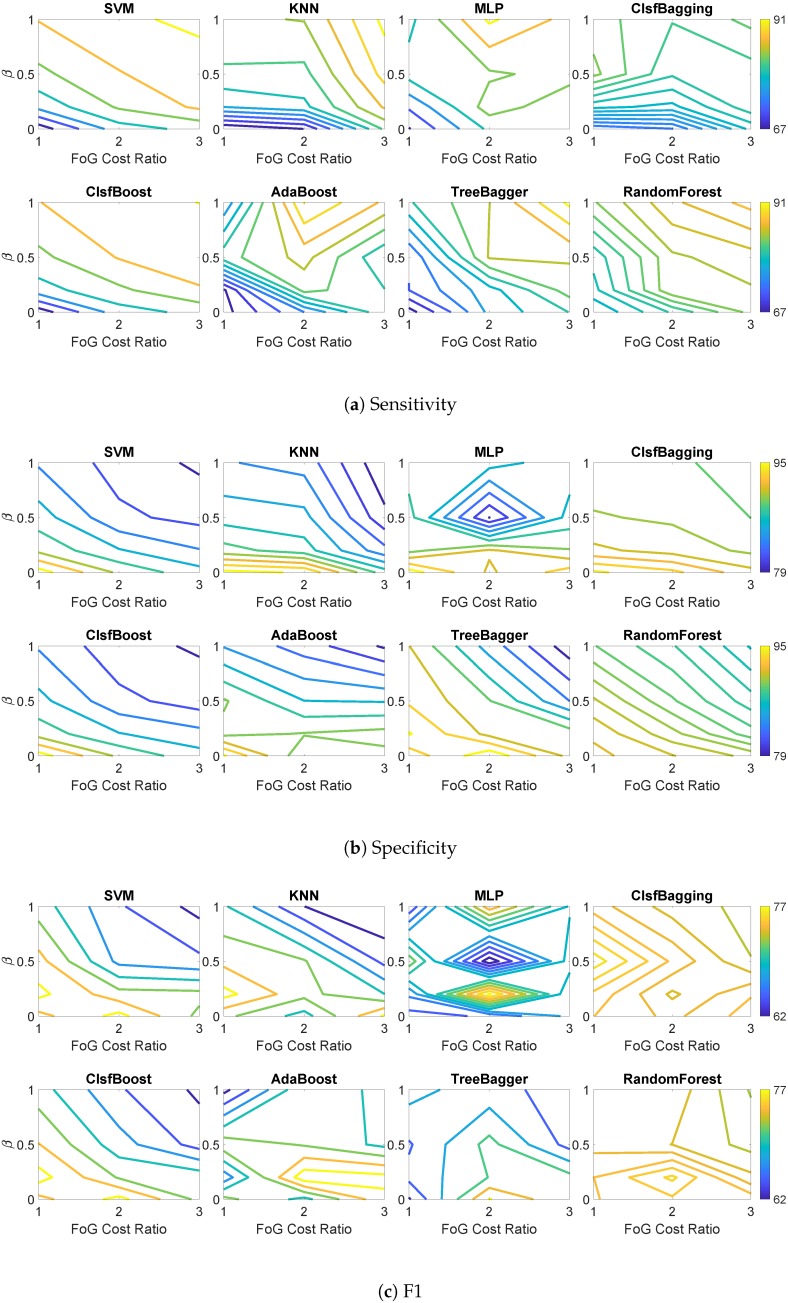
Performance measures of classifiers for patient-independent models using synthetic data and cost of misclassification.

The offline FoG detection system was also evaluated using leave-one-patient-out cross validation (LOOCV). We used data from the same set of participants in patient-dependent models to train classifiers on data from six participants and then test on the remaining participant. Table 5 shows the average performance of patient independent models using the original imbalanced dataset. Among all the individual and ensemble classifiers, RandomForest showed highest sensitivity (77.0%), and ClsfBagging demonstrated highest specificity and F1 (94.2% and 74.5%, respectively). Figure 4 shows how the level of imbalance and ratio of cost affect sensitivity, specificity, and F1 of classifiers in detecting FoG. Table 6 shows the average performance of classifiers and their best results for the three performance measures in patient-independent models. Among all classifiers and combinations of β and cost ratios, KNN showed highest sensitivity (90.6%), and ClsfBagging had the highest specificity (94.2%) and total performance measured by F1 (76.3%). The results show that using data synthesis and increased cost of misclassification, improved sensitivity of ClsfBagging from 72.6% to 83.3% and impaired the specificity from 94.2% to 90.1%, which in sum resulted in improved F1 from 74.5% to 76.3%.

### 3.3. FoG Detection Latency

This section presents the results for FoG detection latency in patient dependent models as it showed higher performance (see Table 4 and Table 6). We refer to latency as the time between a FoG episode onset and the time when the system detects it. As the system runs the FoG detection algorithm every 0.5 s (step time in windowing the acceleration signal), the latency is also observed in steps of 0.5 s. A negative latency represents prediction of FoG (before its actual occurrence) and a positive latency represents detection (after its occurrence). We also assumed that delays caused by sensor data transmission are small and can be neglected.

**Figure 5:**
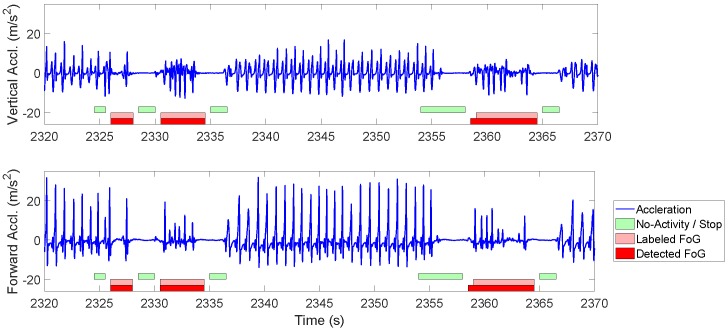
Acceleration signal collected from the right ankle sensor and the corresponding labeled and detected events using of ClsfBagging with β=0.2 and $C_{FoG}=2$.

**Figure 6:**
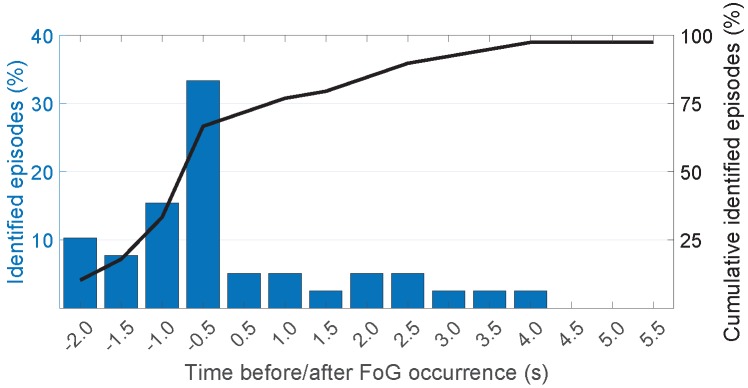
Average FoG detection latency of ClsfBagging in patient-dependent models with β=0.2 and $C_{FoG}=2$. Negative values of time represent duration before FoG onset.

We chose the first 60% of the data from each participant for training and the rest for classifier evaluation. Here we only present the results for ClsfBagging ($\beta =0.2,\;C_{FoG}=2$) as it showed the highest F1 among classifiers in patient-dependent models. Figure 5 depicts the vertical and forward acceleration signals collected from the sensor placed on right ankle of one participant as well as the labeled and detected events. Figure 6 shows the average detection latency of the ClsfBagging classifier for the seven selected patients. The classifier predicted 10.3%, 7.7%, 15.4% and 33.3% (66.7% in total) of the FoG episodes, respectively, 2 s, 1.5 s, 1 s and 0.5 s before the actual FoG occurrence, and detected 30.8% of the episodes within 4 s after FoG onset. The results shown in Figure 6 suggest that ClsfBagging is capable of detecting FoG events within 0.118 ± 1.587 s after FoG onset.

## 4. Discussion

This study investigated the impact of adding new samples to the minority class using data synthesis methods and increased misclassification cost in favor of the minority class on an imbalanced dataset consisting of FoG and normal walking in patients with PD. The system takes time series signals as inputs and extracts features from 2 s windows; the features from six successive windows are then fed to the classifier. Classifier performance results presented in Figure 3 and Figure 4 suggest that adding more synthetic samples to the training set (higher values of β) can shift classification bias in an imbalanced dataset toward the minority class and increase FoG detection sensitivity. It also results in more incorrectly recognized FoG instances, causing specificity to deteriorate. The increased number of true positives and false positives reduces F1 in most cases. The FoG class cost also affects the performance measures in the same way as new synthetic data ratio (i.e., higher cost classifies more instances as FoG resulting in increased sensitivity and reduced specificity and F1 in most cases).

In this study we labeled two windows prior to each freezing onset as FoG in order to enable the system to predict FoG before its actual occurrence. The system was able to predict 66.7% of FoG episode within 2 s before FoG onset. This method improved FoG detection latency (0.118±1.587 s) compared with [37], another study using ensemble classifiers (1.085±0.731 s). Palmerini et al. also discriminated pre-FoG episodes from normal gait after removing data corresponding to FoG and with no sufficient motion. Using patient-dependent training and testing, their method predicted 83% of the FoG episodes within 2 s before their onset [40]. This comparison suggests that using different classifiers to discriminate FoG from prior normal gait or stop states may further improve detection accuracy.

The ensemble ClsfBagging classifier trained in this study outperforms the weighted SVM classifier in [36] (90% and 92% for sensitivity and specificity, respectively). ClsfBagging also performs equally with the deep learning based model in [38] for detecting FoG episodes (90.8% vs. 91.9% sensitivity), but shows fewer false positives (95.0% vs. 89.5% specificity). However, labeling pre-FoG samples as FoG might have resulted in lower sensitivity and specificity when compared to [37] using ensemble classifiers (99.54% and 99.96%, respectively) and [39] using a deep convolutional neural network (99.64% and 99.99%, respectively).

Also, in patient-independent models, ClsfBagging detected more FoG episodes (83.3% vs. 66.3% sensitivity) but also showed more false positives (90.1% vs 95.4% specificity) compared with [37]. ClsfBagging also outperforms the deep convolutional neural network in [39] (74.43% and 90.60% sensitivity and specificity) which suggests that the proposed model in this study is more generalized in detecting FoG in patients with PD.

For clinical use, the proposed ClsfBagging model (an ensemble classifier formed by SVM, KNN and MLP individual classifiers and trained using bagging methods) will be integrated into an Android application which triggers vibrotactile cueing via a connected smartwatch. The system’s ability to detect FoG episodes, as well as the effects of cueing in ambient environments, will be investigated in future studies. However, considering that the most accurate performance was obtained using patient-dependent models, practical implementation will require data collection from each user in order to train an offline model using expert-labeled signals. A pre-trained model can also be updated using the new data and transfer learning techniques [41,53].

There are two main limitations for the presented study: (1) Among the eighteen participants recruited, only nine froze during the test, two of whom with low number and duration of FoG episodes. This caused an imbalanced dataset with a small sample size to train and test classifiers. A larger pool of participants is required for further analysis of the results. (2) In a clinical setting and to prevent freezing, most of the patients were too focused on walking during the experiments, while they are normally distracted by other tasks in their homes. A set of data collected from participants performing dual-tasks in ambient environments would represent their behaviour more realistically and a model trained on such data would be able to detect FoG episodes more effectively.

## 5. Conclusions

The current work investigated the effect of synthesizing new samples and increased misclassification cost for the minority class on classification accuracy of a binary dataset using individual and ensemble classifiers. The results suggest that using ADASYN to create new samples for the FoG class and increasing its cost shift classification boundaries towards the majority class in the imbalanced dataset. This results in improved recognition of actual freezing instances (sensitivity) and increased incorrectly FoG identified samples (false positive) and reduces specificity and F1. However, in few cases, using synthesized training dataset and unequal cost of misclassification improved F1 in the ensemble classifiers. The results also demonstrate that using ensemble classifiers improved performance of individual classifiers.

In addition to the accuracy of detecting FoG episodes, the latency of detection is also important for external cueing applications. The ideal system would be able to predict imminent FoG episodes and trigger preemptive cueing which can potentially prevent the episodes. The proposed model in this study was able to identify 97.4% of the FoG labeled samples within 2 s before to 4 s after FoG onset, 66.7% of which were predicted in the patient-dependent models.

The classifiers investigated here performed better on patient-dependent models (as compared to patient-independent models). This implies that the future studies on real-time FoG detection must implement an offline training process in which data will be collected and models will be trained as a pre-processing stage. Transfer learning techniques can also be used to update the already trained models using the data collected from each user. Future studies will be also focused on validating the results in ambient settings.

## 6. Future Works

This study demonstrated FoG prediction capability of the patient-dependent models. However, training specific models for each individual may not be practical as each individual freezes more frequently under certain conditions. This inter-subject variability of results in varied class imbalance in the dataset and, consequently, performance of the patient-dependent models. To study the effects of inter-subject and inter-trial variability, the models can be trained and tested on a dataset containing more participants performing more varied tasks.

## Figures and Tables

**Table 1:** Set of features extracted from each window of data.

Feature	Description
Freeze Index	The power in freeze band (3–8 Hz) divided by the power in locomotion band (0.5–3 Hz) using FFT of the acceleration signal [34]
Sample Entropy	The negative logarithm of the ratio of conditional probability of data subsets of length *m* matching point-wise within a tolerance *r*, to conditional probability of data subsets of length m+1 being within the same tolerance [47]
Power	Total power in the freeze and locomotion bands (0.5–8 Hz) of the signal
Standard Deviation	Mean deviation of data points compared to the average

**Table 2:** Confusion matrix (NG: normal gait, FoG: freezing of gait).

		Predicted	
		NG	FoG
**Actual**	**NG**	True Negative (TN)	False Positive (FP)
	**FoG**	False Negative (FN)	True Positive (TP)

**Table 3:** Performance of classifiers in patient dependent models using the original imbalanced dataset and equal cost of misclassification (Results show average performance of all seven participants). The bold values show the highest performance among all the classifiers.

Classifier	Sensitivity (%)	Specificity (%)	F1 (%)
SVM	**85.6**	92.3	84.9
KNN	82.0	95.3	86.0
MLP	82.4	94.5	85.4
ClsfBagging	85.2	**95.8**	**87.7**
ClsfBoost	85.1	94.2	85.8
AdaBoost	82.0	94.6	82.8
TreeBagger	83.9	95.4	85.8
RandomForest	82.9	95.4	85.6

**Table 4:** Best performing classifiers in patient dependent models (Results show average performance of all seven participants). The bold values show the highest performance among all the classifiers.

Classifier	$C_{NG}$	$C_{FoG}$	β	Sensitivity (%)	Specificity (%)	F1 (%)
KNN	1	3	1	**97.6**	88.6	83.4
KNN	1	1	0	78.5	**96.2**	83.9
ClsfBagging	1	2	0.2	90.8	95.0	**90.7**
ClsfBagging	1	1	0.2	90.5	95.5	**90.5**
ClsfBagging	1	2	0.5	90.2	94.7	**89.7**

**Table 5:** Performance of classifiers in patient independent models using the original imbalanced dataset and equal cost of misclassification (Results show average performance of all seven participants). The bold values show the highest performance among all the classifiers.

Classifier	Sensitivity (%)	Specificity (%)	F1 (%)
SVM	76.9	87.5	71.3
KNN	68.2	93.3	69.6
MLP	67.9	89.8	63.8
ClsfBagging	72.1	**94.2**	**74.5**
ClsfBoost	76.9	87.5	71.3
AdaBoost	73.8	92.6	72.2
TreeBagger	76.5	90.2	72.9
RandomForest	**77.0**	91.7	**74.5**

**Table 6:** Best performing classifiers in patient independent models (Results show average performance of all seven participants). The bold values show the highest performance among all the classifiers.

Classifier	$C_{NG}$	$C_{FoG}$	β	Sensitivity (%)	Specificity (%)	F1 (%)
KNN	1	3	1	**90.6**	82.2	67.0
ClsfBagging	1	1	0	72.1	**94.2**	74.5
ClsfBagging	1	1	0.5	83.3	90.1	**76.3**
RandomForest	1	2	0.2	83.8	90.2	**75.6**
TreeBagger	1	2	0	81.3	91.4	**75.5**

## Abbreviations

The following abbreviations are used in this manuscript:

ADASYN	Adaptive Synthetic
Bagging	Bootstrap Aggregating
Clsf	Classifiers
DA	Dopamine agonist
DBS	Deep Brain Stimulation
DL	Deep Learning
EEG	Electrocardiography
EMG	Electromyography
FN	False Negative
FoG	Freezing of Gait
FOGQ	Freezing of Gait Questionnaire
FP	False Positive
IMU	Inertial Measurement Unit
IRB	Institutional Review Board
KNN	K-Nearest Neighbor
LD	Levodopa
MLP	Multi Layer Perceptron
RAS	Rhythmic Auditory Stimulation
SMOTE	Synthetic Minority Over-Sampling Technique
SVM	Support Vector Machine
TN	True Negative
TP	True Positive

## References

[1] Boonstra T.A., Van Der Kooij H., Munneke M., Bloem B.R. (2008). Gait disorders and balance disturbances in Parkinson’s disease: clinical update and pathophysiology. Curr. Opin. Neurol..

[2] Muslimović D., Post B., Speelman J.D., Schmand B., de Haan R.J. (2008). Determinants of disability and quality of life in mild to moderate Parkinson disease. Neurology.

[3] DeMaagd G., Philip A. (2015). Parkinson’s Disease and Its Management: Part 1: Disease Entity, Risk Factors, Pathophysiology, Clinical Presentation, and Diagnosis. Pharm. Ther..

[4] Nazifi M.M., Yoon H.U., Beschorner K., Hur P. (2017). Shared and task-specific muscle synergies during normal walking and slipping. Front. Hum. Neurosci..

[5] Nevisipour M., Grabiner M.D., Honeycutt C.F. (2019). A single session of trip-specific training modifies trunk control following treadmill induced balance perturbations in stroke survivors. Gait Posture.

[6] Elkouzi A., Bit-Ivan E.N., Elble R.J. (2017). Pure akinesia with gait freezing: A clinicopathologic study. J. Clin. Mov. Disord..

[7] Nutt J.G., Bloem B.R., Giladi N., Hallett M., Horak F.B., Nieuwboer A. (2011). Freezing of gait: Moving forward on a mysterious clinical phenomenon. Lancet Neurol..

[8] Ehgoetz Martens K.A., Ellard C.G., Almeida Q.J. (2014). Does Anxiety Cause Freezing of Gait in Parkinson’s Disease. PLoS ONE.

[9] Spildooren J., Vercruysse S., Desloovere K., Vandenberghe W., Kerckhofs E., Nieuwboer A. (2010). Freezing of gait in Parkinson’s disease: The impact of dual-tasking and turning. Mov. Disord..

[10] Mancini M., Bloem B.R., Horak F.B., Lewis S.J., Nieuwboer A., Nonnekes J. (2019). Clinical and methodological challenges for assessing freezing of gait: Future perspectives. Mov. Disord..

[11] Peterson D.S., King L.A., Cohen R.G., Horak F.B. (2016). Cognitive contributions to freezing of gait in Parkinson disease: implications for physical rehabilitation. Phys. Ther..

[12] Nieuwboer A., de Weerdt W., Dom R., Lesaffre E. (1998). A frequency and correlation analysis of motor deficits in Parkinson patients. Disabil. Rehabil..

[13] Ehgoetz Martens K.A., Pieruccini-Faria F., Almeida Q.J. (2013). Could Sensory Mechanisms Be a Core Factor That Underlies Freezing of Gait in Parkinson’s Disease?. PLoS ONE.

[14] Ahn S., Chen Y., Bredow T., Cheung C., Yu F. (2017). Effects of Non-Pharmacological Treatments on Quality of Life in Parkinson’s Disease: A Review. J. Park. Dis. Alzheimer Dis..

[15] Giladi N. (2008). Medical treatment of freezing of gait. Mov. Disord..

[16] Huang C., Chu H., Zhang Y., Wang X. (2018). Deep Brain Stimulation to Alleviate Freezing of Gait and Cognitive Dysfunction in Parkinson’s Disease: Update on Current Research and Future Perspectives. Front. Neurosci..

[17] Xie T., Vigil J., MacCracken E., Gasparaitis A., Young J., Kang W., Bernard J., Warnke P., Kang U.J. (2015). Low-frequency stimulation of STN-DBS reduces aspiration and freezing of gait in patients with PD. Neurology.

[18] Fitts P.M., Posner M.I. (1967). Learning and Skilled Performance in Human Performance.

[19] Ginis P., Nackaerts E., Nieuwboer A., Heremans E. (2018). Cueing for people with Parkinson’s disease with freezing of gait: A narrative review of the state-of-the-art and novel perspectives. Ann. Phys. Rehabil. Med..

[20] Peterson D.S., Smulders K. (2015). Cues and Attention in Parkinsonian Gait: Potential Mechanisms and Future Directions. Front. Neurol..

[21] Ghai S., Ghai I., Schmitz G., Effenberg A.O. (2017). Effect of rhythmic auditory cueing on parkinsonian gait: A systematic review and meta-analysis. Aging Dis..

[22] Lim I., van Wegen E., de Goede C., Deutekom M., Nieuwboer A., Willems A., Jones D., Rochester L., Kwakkel G. (2005). Effects of external rhythmical cueing on gait in patients with Parkinson’s disease: A systematic review. Clin. Rehabil..

[23] Frazzitta G., Maestri R., Uccellini D., Bertotti G., Abelli P. (2009). Rehabilitation treatment of gait in patients with Parkinson’s disease with freezing: A comparison between two physical therapy protocols using visual and auditory cues with or without treadmill training. Mov. Disord..

[24] Nieuwboer A., Kwakkel G., Rochester L., Jones D., Van Wegen E., Willems A.M., Chavret F., Hetherington V., Baker K., Lim I. (2007). Cueing training in the home improves gait-related mobility in Parkinson’s disease: The RESCUE trial. J. Neurol. Neurosurg. Psychiatry.

[25] Kadivar Z., Corcos D.M., Foto J., Hondzinski J.M. (2011). Effect of Step Training and Rhythmic Auditory Stimulation on Functional Performance in Parkinson Patients. Neurorehabilit. Neural Repair.

[26] Cubo E., Leurgans S., Goetz C.G. (2004). Short-term and practice effects of metronome pacing in Parkinson’s disease patients with gait freezing while in the ‘on’ state: randomized single blind evaluation. Park. Relat. Disord..

[27] Mazilu S., Calatroni A., Gazit E., Mirelman A., Hausdorff J.M., Tröster G. (2015). Prediction of Freezing of Gait in Parkinson’s From Physiological Wearables: An Exploratory Study. IEEE J. Biomed. Health Inf..

[28] Nieuwboer A., Dom R., De Weerdt W., Desloovere K., Janssens L., Stijn V. (2004). Electromyographic profiles of gait prior to onset of freezing episodes in patients with Parkinson’s disease. Brain.

[29] Cole B.T., Roy S.H., Nawab S.H. Detecting freezing-of-gait during unscripted and unconstrained activity. Proceedings of the 2011 Annual International Conference of the IEEE Engineering in Medicine and Biology Society.

[30] Koh S.B., Park K.W., Lee D.H., Kim S.J., Yoon J.S. (2008). Gait Analysis in Patients With Parkinson’s Disease: Relationship to Clinical Features and Freezing. J. Mov. Disord..

[31] Delval A., Snijders A.H., Weerdesteyn V., Duysens J.E., Defebvre L., Giladi N., Bloem B.R. (2010). Objective detection of subtle freezing of gait episodes in Parkinson’s disease. Mov. Disord..

[32] Plotnik M., Giladi N., Balash Y., Peretz C., Hausdorff J.M. (2005). Is freezing of gait in Parkinson’s disease related to asymmetric motor function?. Ann. Neurol..

[33] Hausdorff J.M., Schaafsma J.D., Balash Y., Bartels A.L., Gurevich T., Giladi N. (2003). Impaired regulation of stride variability in Parkinson’s disease subjects with freezing of gait. Exp. Brain Res..

[34] Moore S.T., MacDougall H.G., Ondo W.G. (2008). Ambulatory monitoring of freezing of gait in Parkinson’s disease. J. Neurosci. Methods.

[35] Tripoliti E.E., Tzallas A.T., Tsipouras M.G., Rigas G., Bougia P., Leontiou M., Konitsiotis S., Chondrogiorgi M., Tsouli S., Fotiadis D.I. (2013). Automatic detection of freezing of gait events in patients with Parkinson’s disease. Comput. Methods Programs Biomed..

[36] Tahafchi P., Molina R., Roper J.A., Sowalsky K., Hass C.J., Gunduz A., Okun M.S., Judy J.W. Freezing-of-Gait detection using temporal, spatial, and physiological features with a support-vector-machine classifier. Proceedings of the 2017 39th Annual International Conference of the IEEE Engineering in Medicine and Biology Society (EMBC).

[37] Mazilu S., Hardegger M., Zhu Z., Roggen D., Troester G., Plotnik M., Hausdorff J. Online Detection of Freezing of Gait with Smartphones and Machine Learning Techniques. Proceedings of the 6th International Conference on Pervasive Computing Technologies for Healthcare.

[38] Camps J., Samà A., Martín M., Rodríguez-Martín D., Pérez-López C., Arostegui J.M.M., Cabestany J., Català A., Alcaine S., Mestre B. (2018). Deep learning for freezing of gait detection in Parkinson’s disease patients in their homes using a waist-worn inertial measurement unit. Knowl.-Based Syst..

[39] Xia Y., Zhang J., Ye Q., Cheng N., Lu Y., Zhang D. (2018). Evaluation of deep convolutional neural networks for detection of freezing of gait in Parkinson’s disease patients. Biomed. Signal Process. Control..

[40] Palmerini L., Rocchi L., Mazilu S., Gazit E., Hausdorff J.M., Chiari L. (2017). Identification of characteristic motor patterns preceding freezing of gait in Parkinson’s disease using wearable sensors. Front. Neurol..

[41] Torvi V.G., Bhattacharya A., Chakraborty S. Deep Domain Adaptation to Predict Freezing of Gait in Patients with Parkinson’s Disease. Proceedings of the 2018 17th IEEE International Conference on Machine Learning and Applications (ICMLA).

[42] Galar M., Fernandez A., Barrenechea E., Bustince H., Herrera F. (2012). A Review on Ensembles for the Class Imbalance Problem: Bagging-, Boosting-, and Hybrid-Based Approaches. IEEE Trans. Syst. Man Cybern. Part C (Appl. Rev.).

[43] He H., Bai Y., Garcia E.A., Li S. ADASYN: Adaptive synthetic sampling approach for imbalanced learning. Proceedings of the 2008 IEEE International Joint Conference on Neural Networks (IEEEWorld Congress on Computational Intelligence).

[44] Kotsiantis S., Kanellopoulos D., Pintelas P. (2006). Handling imbalanced datasets: A review. Gests Int. Trans. Comput. Sci. Eng..

[45] Schaafsma J.D., Balash Y., Gurevich T., Bartels A.L., Hausdorff J.M., Giladi N. (2003). Characterization of freezing of gait subtypes and the response of each to levodopa in Parkinson’s disease. Eur. J. Neurol..

[46] Naghavi N., Wade E. (2019). Prediction of Freezing of Gait in Parkinson’s Disease Using Statistical Inference and Lower–Limb Acceleration Data. IEEE Trans. Neural Syst. Rehabil. Eng..

[47] Tochigi Y., Segal N.A., Vaseenon T., Brown T.D. (2012). Entropy analysis of tri-axial leg acceleration signal waveforms for measurement of decrease of physiological variability in human gait. J. Orthop. Res..

[48] Chawla N.V., Bowyer K.W., Hall L.O., Kegelmeyer W.P. (2002). SMOTE: Synthetic minority over-sampling technique. J. Artif. Intell. Res..

[49] He H., Garcia E.A. (2009). Learning from Imbalanced Data. IEEE Trans. Knowl. Data Eng..

[50] Dietterich T.G. (2000). Ensemble Methods in Machine Learning. Multiple Classifier Systems.

[51] Topchy A., Jain A.K., Punch W. (2005). Clustering ensembles: models of consensus and weak partitions. IEEE Trans. Pattern Anal. Mach. Intell..

[52] Sarvari H., Domeniconi C., Stilo G. Graph-based Selective Outlier Ensembles. Proceedings of the 34th ACM/SIGAPP Symposium on Applied Computing.

[53] Borhani S., Abiri R., Zhao X., Jiang Y. (2017). A Transfer Learning Approach towards Zero-Training BCI for EEG-Based Two Dimensional Cursor Control.

